# Impact of Maternal Diabetes on the Incidence of Critical Congenital Heart Disease in the United States

**DOI:** 10.1016/j.jacadv.2025.102176

**Published:** 2025-09-18

**Authors:** Karthik Gonuguntla, Mohamed Abugrin, Harshith Thyagaturu, Hafiz Muhammad Waqar Younas, Hardik Valand, Prakash Upreti, Harsh A. Patel, Muchi Ditah Chobufo, Vijaykumar Sekar, Ayesha Shaik, Muhammad Zia Khan, Yasar Sattar, Martha Gulati

**Affiliations:** a Department of Cardiology, West Virginia University, Morgantown, West Virginia, USA; b Department of Internal Medicine, Bassett Medical Center, Cooperstown, New York, USA; c Department of Internal Medicine, Weiss Memorial Hospital, Chicago, Illinois, USA; d Department of Internal Medicine, Trinity Health System, Steubenville, Ohio, USA; e Sands-Constellation Heart Institute, Rochester Regional Health, Rochester, New York, USA; f Geetanjali Medical College and Hospital, Udaipur, Rajasthan, India; g Lehigh Valley Health Network, Allentown, Pennsylvania, USA; h Department of Cardiology, MGH, Boston, Massachusetts, USA; i Barbra Streisand Women’s Heart Center, Smidt Heart Institute, Cedars-Sinai Medical Center, Los Angeles, California, USA; j The Baim Institute for Clinical Research, Boston, Massachusetts, USA

**Keywords:** congenital anomalies, critical congenital heart disease, epidemiology, gestational diabetes, maternal diabetes

## Abstract

**Background:**

Critical congenital heart disease (CCHD) represents a significant subset of congenital heart disease (CHD). While the association between maternal diabetes mellitus and offspring CHD is well established, the specific relationship between maternal diabetes and CCHD remains underexplored.

**Objectives:**

This study aims to investigate the association between maternal diabetes and the incidence of offspring CCHD.

**Methods:**

We analyzed natality data from the Centers for Disease Control and Prevention Wide-Ranging Online Data for Epidemiologic Research (CDC WONDER) from 2016 to 2021. The data set included information on maternal and paternal attributes, pregnancy history, prenatal care, and congenital anomalies among newborns. We included all live births in the United States, focusing on single births at a gestational age of ≥20 weeks. Multivariable logistic regression was used to explore the relationship between gestational diabetes, pregestational diabetes, and CCHD.

**Results:**

Among 22,646,079 live births, 13,533 cases of CCHD were identified, with an incidence of 6 per 10,000 live births. Pregestational diabetes was associated with a 4.33-fold higher risk of CCHD (aOR: 4.33; 95% CI: 3.93-4.76), and gestational diabetes with a 1.47-fold higher risk (aOR: 1.47; 95% CI: 1.38-1.57). Additional risk factors included pregestational hypertension, gestational hypertension, and late initiation of antenatal care. A longer gestational age was associated with a lower risk of CCHD.

**Conclusions:**

Maternal diabetes, both pregestational and gestational, significantly increases the risk of CCHD. These findings highlight the need for targeted interventions and monitoring of diabetic mothers to mitigate the risk of CCHD in their offspring.

Congenital heart disease (CHD) refers to structural abnormalities or malformations of the great vessels during fetal development. Critical congenital heart disease (CCHD) represents a subtype of CHD, comprising 15 to 20% of all cases.^1^ Asia and Europe exhibit a higher prevalence of CCHD compared to North America, with low-income countries experiencing elevated CCHD-related mortality rates.^2^

CCHD cases are often sporadic but can also be associated with genetic syndromes. Approximately 15 to 20% of infants diagnosed with CCHD have known chromosomal abnormalities.^3^ Factors associated with CCHD, including maternal diabetes, illness, phenylketonuria, and maternal exposure to toxins, drugs, or viral infections during the peripartum period.^3^

The prevalence of type 2 diabetes mellitus has been rising significantly in the United States. According to the Centers for Disease Control and Prevention (CDC), 38.4 million people in the United States have diabetes, and 97.6 million people aged 18 years or older have prediabetes.^4^ Slightly less than one percent of pregnancies have established prepregnancy diabetes,^5^ and almost 8% of pregnancies are affected by gestational diabetes, with this number rising annually.^6^ The association between diabetes mellitus and CHD is well established, with several proposed pathophysiological mechanisms explaining this link.^7^, ^8^, ^9^, ^10^, ^11^ Many of these mechanisms are driven by maternal hyperglycemia, which plays a significant role in the development of CHD.

Despite the established association between diabetes and CHD, the relationship between CCHD and diabetes is yet to be thoroughly explored. To bridge this knowledge gap, this study aims to investigate the association between CCHD and maternal diabetes.

## Methods

Our analysis utilized natality data sourced from the Centers for Disease Control and Prevention Wide-Ranging Online Data for Epidemiologic Research (CDC WONDER). This data set offers a comprehensive range of information, including details on maternal and paternal attributes, pregnancy history, prenatal care specifics, risk factors during pregnancy, maternal infections, delivery attributes, infant characteristics, and congenital anomalies among newborns, among other factors. This data set categorizes congenital heart defects under broader groupings rather than listing each condition individually. The CCHD category includes conditions such as tetralogy of Fallot, transposition of the great arteries, tricuspid atresia, pulmonary atresia, total anomalous pulmonary venous return, truncus arteriosus, and hypoplastic left heart syndrome. From 2016 to 2021, we conducted a thorough examination of maternal and fetal records encompassing all live births in the United States. Our study adhered to established definitions for gestational diabetes, prepregnancy diabetes, and gestational and pregestational hypertension as per prevailing standards. All data are publicly available and can be accessed at https://wonder.cdc.gov/natality.html.

Maternal smoking status, race, level of education, and father’s age were provided through self-reporting. Information regarding the birth, such as the newborn's sex, birth weight, gestational age, plurality, and any congenital anomalies, was reported by the pregnancy care team. Education level was categorized into 3 groups: high school or below, any college education, and a master's degree or higher. Race was classified into 4 groups: non-Hispanic White, non-Hispanic Black, Hispanic, and all other races grouped as “other.” Infection complications included any reported cases of syphilis, gonorrhea, chlamydia, hepatitis B, or hepatitis C that were diagnosed or treated during pregnancy. National birth certificate records on heart diseases solely included cyanotic heart lesions diagnosable within the first 24 hours of life, excluding subtypes. The analysis included single births with a gestational age of ≥20 weeks at the time of delivery.

Categorical variables were presented as n (%) while continuous variables were expressed as median (IQR). Comparisons between groups were conducted using the chi-square test for categorical variables and Student’s *t*-test for normally distributed continuous variables, or the Mann-Whitney *U* test for non-normally distributed continuous variables. Multivariable logistic regression analysis was used to explore the relationship between gestational diabetes and prediabetes with CCHD. Only factors that enhanced the model fit or were recognized as established risk factors for CCHD or other CHDs were retained in the final model. Model fit and effect modification were assessed using the likelihood ratio test. Chi-square tests for trend and heterogeneity were utilized to evaluate trends (in continuous variables and ordinal categorical variables) and deviations from a linear trend. All analyses were performed using Stata v17, with *P* values below 0.05 considered statistically significant.

The CDC WONDER data set, provided by the CDC, is publicly accessible and contains deidentified information. Because it lacks any identifiable individual data, ethical research standards dictate that secondary data analysis of such publicly available data does not require Institutional Review Board approval. Our study aligns with these principles by solely utilizing the deidentified and publicly available CDC WONDER data set.

## Results

The primary cohort comprised 22,646,079 live births between 2016 and 2021. The incidence of CCHD from 2016 to 2021 was 6 per 10,000 live births, with 13,533 unique cases. Of these, 7,426 were male and 6,119 were female. The incidence of CCHD remained stable throughout the data collection period, with no statistically significant change from 2016 to 2021. The median (IQR) for body mass index, birthweight, gestational age, maternal age, and paternal age were 27.2 kg/m^2^ (22.3-30.8), 3,299 g (2,960-3,629), 39 weeks (38-40), 29 years (25-33), and 31 years (27-36), respectively. Additional demographic and clinical characteristics of the study cohort stratified by diabetes status are detailed in Table 1.

**Table 1:** Demographic and Clinical Characteristics of the Study Cohort Stratified by Diabetes Status

	No Diabetes (n = 20,848,588)	Pregestational Diabetes (n = 219,451)	Gestational Diabetes (n = 1,578,040)	*P* Value∗
Maternal age, y	29.0 (25.0-33.0)	31.0 (27.0-36.0)	32.0 (28.0-35.0)	<0.001
Paternal age, y	31.0 (27.0-36.0)	33.0 (29.0-38.0)	33.0 (29.0-38.0)	<0.001
Maternal smoking	1,646,404 (7.9%)	20,759 (9.5%)	119,717 (7.6%)	<0.001
First trimester smoking	1,246,396 (6.0%)	15,948 (7.3%)	85,332 (5.4%)	<0.001
Pregestational hypertension	377,775 (1.8%)	37,724 (17.2%)	78,773 (5.0%)	<0.001
Gestational hypertension	1,439,715 (6.9%)	37,516 (17.1%)	210,046 (13.3%)	<0.001
BMI	25.5 (22.1-30.3)	31.8 (26.5-38.1)	29.3 (24.7-35.1)	<0.001
Eclampsia	52,310 (0.3%)	2,006 (0.9%)	6,928 (0.4%)	<0.001
Birthweight (kg)	3.29 (2.96-3.62)	3.3 (2.84-3.74)	3.3 (2.94-3.65)	<0.001
Gestational age (wk)	39 (38-40)	38 (36-39)	39 (37-39)	<0.001
Maternal level of education				<0.001
High school or less	7,910,698 (38.5%)	95,412 (44.0%)	570,296 (36.7%)	
College education	10,077,569 (49.0%)	102,529 (47.3%)	785,433 (50.5%)	
Master’s and above	2,575,997 (12.5%)	18,827 (8.7%)	199,883 (12.8%)	
Assisted reproductive technology^a^	242,867 (1.2%)	3,178 (1.5%)	33,173 (2.1%)	0.158
Fertility drugs^b^	153,559 (0.7%)	2,236 (1.0%)	20,675 (1.3%)	0.129
Infertility treatment^c^	387,429 (1.9%)	5,398 (2.5%)	52,789 (3.3%)	<0.001
Multiple gestation	675,271 (3.2%)	8,071 (3.7%)	68,926 (4.4%)	<0.001
Congenital infections	578,098 (2.8%)	6,187 (2.8%)	36,437 (2.3%)	<0.001
Sex of baby				<0.001
Male	10,653,447 (51.1%)	112,436 (51.2%)	816,732 (51.8%)	
Female	10,195,141 (48.9%)	107,015 (48.8%)	761,308 (48.2%)	
Maternal race				<0.001
NH White	10,826,254 (51.9%)	91,272 (41.6%)	729,236 (46.2%)	
NH Black	3,040,022 (14.6%)	42,767 (19.5%)	183,862 (11.7%)	
Hispanic	4,913,252 (23.6%)	58,977 (26.9%)	405,832 (25.7%)	
Other	2,069,060 (9.9%)	26,435 (12.0%)	259,110 (16.4%)	
Birth order				<0.001
First live delivery	7,991,338 (38.4%)	73,279 (33.5%)	522,168 (33.2%)	<0.001
2-4 previous deliveries	11,713,125 (56.3%)	128,447 (58.7%)	941,778 (59.8%)	<0.001
≥5 previous live deliveries	1,087,706 (5.2%)	17,249 (7.9%)	111,221 (7.1%)	<0.001
Prior pregnancy terminations^d^				<0.001
0	15,229,917 (73.3%)	138,727 (63.4%)	1,056,617 (67.1%)	
1-2	4,858,979 (23.4%)	65,939 (30.1%)	442,194 (28.1%)	
≥3	695,821 (3.3%)	14,231 (6.5%)	75,983 (4.8%)	

BMI = body mass index; N = number; NH = non-Hispanic.

∗ *P* value compares no diabetes and diabetes group (including pregestational and gestational).

a Assisted reproductive technology includes in vitro fertilization (IVF) or gamete intrafallopian transfer (GIFT).

b Fertility drugs were considered in the data set; however, specific drugs were not detailed, only whether fertility-enhancing drugs were used or not.

c Infertility treatment includes the use of fertility-enhancing drugs, artificial insemination or intrauterine insemination, and assisted reproductive technology (eg, IVF, GIFT).

d Prior pregnancy terminations refer to the number of previous pregnancies that ended in either induced abortion or miscarriage.

The prevalence of pregestational diabetes in the study sample was 1.0% and that of gestational diabetes was 7.0%. The prevalence of pregestational diabetes increased from 0.86% in 2016 to 1.08% in 2021, whereas that of gestational diabetes increased from 5.95% to 8.29% over the same period (*P* < 0.001). The annual distribution of births by maternal diabetes status is presented in Table 2.

**Table 2:** Annual Distribution of Births by Maternal Diabetes Status in Different Years

Year	No Diabetes	Pregestational DM	Gestational DM	All
2016	3,683,247 (93.19%)	33,856 (0.86%)	235,155 (5.95%)	3,952,258 (100%)
2017	3,580,558 (92.74%)	35,365 (0.92%)	245,045 (6.35%)	3,860,968 (100%)
2018	3,509,928 (92.40%)	35,770 (0.94%)	252,863 (6.66%)	3,798,561 (100%)
2019	3,458,451 (92.12%)	36,603 (0.98%)	259,078 (6.90%)	3,754,132 (100%)
2020	3,295,455 (91.15%)	38,105 (1.05%)	282,039 (7.80%)	3,615,599 (100%)
2021	3,320,949 (90.62%)	39,752 (1.08%)	303,860 (8.29%)	3,664,561 (100%)

DM = diabetes mellitus; percentages represent the proportion of total cases for each year.

In multivariable models, a significantly higher likelihood of CCHD was observed in infants born to mothers with pregestational diabetes (aOR: 4.33; 95% CI: 3.93-4.76; *P* < 0.001) and gestational diabetes (aOR: 1.47; 95% CI: 1.38-1.57; *P* < 0.001). Other factors associated with an increased risk included pregestational hypertension (aOR: 1.34; 95% CI: 1.21-1.47; *P* < 0.001), gestational hypertension (aOR: 1.16; 95% CI: 1.08-1.23; *P* < 0.001), maternal smoking (aOR: 1.18; 95% CI: 1.05-1.32; *P* = 0.016), low birthweight (aOR: 3.90; 95% CI: 3.73-4.08; *P* < 0.001), infants of non-Hispanic White mothers (aOR: 1.88; 95% CI: 1.76-2.01; *P* < 0.001), and late initiation of antenatal care (relative risk: 1.55; 95% CI: 1.50-1.59; *P* < 0.001). Infection during pregnancy and eclampsia were not statistically significant (aOR: 1.11; 95% CI: 0.98-1.26; *P* = 0.108) and (aOR: 1.73; 95% CI: 0.71-4.21; *P* = 0.224), respectively.

Conversely, a lower likelihood of CCHD was observed in infants of mothers with longer gestational ages (relative risk: 0.91; 95% CI: 0.89-0.94; *P* < 0.001). Additional covariates included in our multivariate analysis are presented in Table 3.

**Table 3:** Adjusted Odds of Covariates Associated With Cyanotic Congenital Heart Disease

	Odds Ratio (95% CI)	*P* Value
Maternal age (per 5 y)	1.08 (1.05-1.11)	<0.001
Paternal age (per 5 y)	0.99 (0.97-1.01)	0.358
Male sex	1.26 (1.21-1.31)	<0.001
Pregestational diabetes	4.33 (3.93-4.76)	<0.001
Gestational diabetes	1.47 (1.38-1.57)	<0.001
Pregestational hypertension	1.34 (1.21-1.47)	<0.001
Gestational hypertension	1.16 (1.08-1.23)	<0.001
Eclampsia	1.73 (0.71-4.21)	0.224
Gestational age (per month)	0.91 (0.89-0.94)	<0.001
Low birthweight	3.90 (3.73-4.08)	<0.001
BMI (per 5 units)	1.04 (1.01-1.08)	0.003
Maternal smoking	1.18 (1.05-1.32)	0.016
Congenital infections	1.11 (0.98-1.26)	0.113
Previous preterm delivery	1.31 (1.21-1.42)	<0.001
Assisted reproductive technology	0.99 (0.81-1.21)	0.958
Late ANC (per month)^a^	1.55 (1.50-1.59)	<0.001
Educational level		
High school or less	Reference	
College education	1.07 (1.02-1.12)	0.007
Master’s and above	0.98 (0.91-1.05)	0.525
Maternal ethnicity		
Black	Reference	
NH Whites	1.88 (1.76-2.01)	<0.001
Hispanics	1.02 (0.94-1.10)	0.673
Others	1.11 (1.01-1.21)	0.028
Birth order		
1	Reference	
2-4	1.13 (1.09-1.18)	<0.001
≥5	1.40 (1.28-1.53)	<0.001

ANC = antenatal care; other abbreviations as in Table 1.

The model was adjusted for maternal age, paternal age, infant sex, pregestational diabetes, gestational diabetes, pregestational hypertension, gestational hypertension, eclampsia, gestational age, low birth weight, body mass index (BMI), maternal smoking, congenital infection, previous preterm delivery, assisted reproductive technology, timing of the first antenatal care (ANC) visit, education status, maternal ethnicity, and birth order.

a Late initiation of ANC is associated with increased risk.

## Discussion

In our study of over 22 million births over a period of 6 years, we found a significant association between CCHD and the presence of pregestational diabetes and gestational diabetes. We found that the incidence of CCHD was 6 per 10,000 live births; however, the presence of pregestational diabetes was associated with a 4.33-fold higher risk of CCHD, whereas the presence of gestational diabetes was associated with a 1.47-fold higher risk. Male infants and older infants, White mothers with higher body mass indexes, a history of preterm delivery, and late antenatal care were at a significantly higher risk of CCHD. Higher gestational age, birth weight, and maternal smoking were associated with a lower risk of CCHD. Additionally, the prevalence of pregestational diabetes has increased by 0.22%, and the incidence of gestational diabetes has increased by 2.34% from 2016 to 2021.

The connection between maternal diabetes and CHD is well established (Central Illustration).^12^ Most studies have assessed the relationship between diabetes and CHD as a whole, possibly due to the lower incidence of CCHD. However, our population-level study specifically explored the association between maternal diabetes and CCHD. Our findings revealed a significant increase in risk, showing a more than four-fold elevation in the incidence of CCHD in infants born to mothers with pregestational diabetes. This aligns with the existing literature that similarly indicates an increased risk between pregestational diabetes and various subtypes of CHD. Certain studies suggest a particularly high risk for conotruncal and laterality abnormalities in infants of diabetic mothers^12^^,^^13^. These studies may help explain the comparable risks observed between maternal diabetes and offspring with CHD and what we have demonstrated in those with maternal diabetes and this association with CCHD. Experimentally supported pathophysiological mechanisms that might alter cardiac development have been proposed, including glucose-mediated disturbances of left-right patterning,^7^ increased apoptosis due to oxidative or other cellular stress,^8^^,^^9^ deficiencies in nitric oxide signaling,^14^ impaired autophagy,^10^ and alterations in neural crest cell formation and migration.^11^

**Central Illustration:**
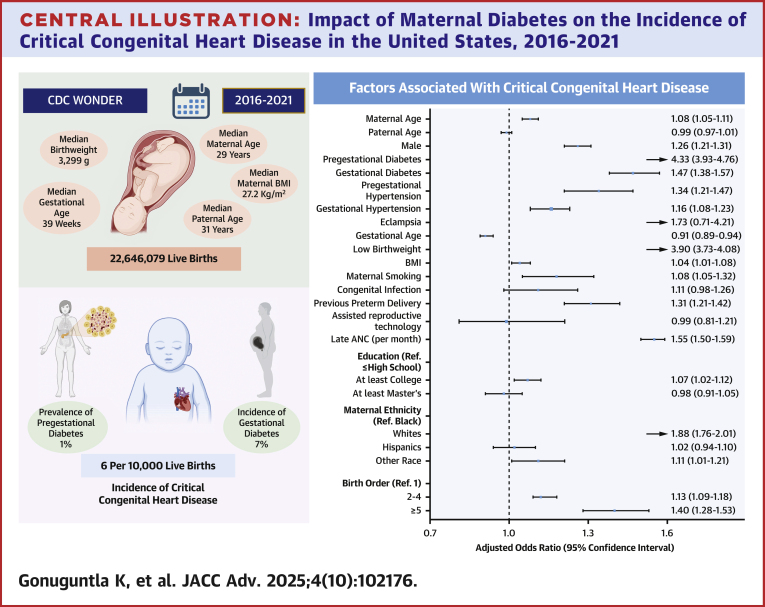
Impact of Maternal Diabetes on the Incidence of Critical Congenital Heart Disease in the United States, 2016-2021 Pregestational and gestational diabetes were significantly associated with an increased risk of critical congenital heart disease (CCHD) in offspring, highlighting the need for stringent glycemic control, comprehensive preconception counseling, and meticulous glucose management throughout pregnancy. The forest plot shows the factors/covariates associated with the presence of CCHD. These findings emphasize the importance of targeted preventive strategies and early intervention in women with pregestational or gestational diabetes to mitigate adverse cardiovascular outcomes in newborns. ANC = antenatal care; BMI = body mass index; Ref. = reference.

Studies examining the association between gestational diabetes and CHD demonstrate conflicting results^15^, ^16^, ^17^ and their relationship is less clear. Our study indicates a 1.47-fold increase in the risk of CCHD in the infants of mothers with gestational diabetes. The lower increase in risk compared to pregestational diabetes may be because cardiac development primarily occurs during the first trimester and is typically complete by the sixth week of pregnancy. Despite the completion of cardiac development in the first trimester, gestational diabetes, usually diagnosed later in pregnancy (second and third trimester), still appears to be an independent risk factor for CCHD in this analysis. The pathophysiological mechanisms underlying the association between gestational diabetes and CCHD may include increased insulin resistance, leading to elevated inflammatory markers, dyslipidemia, and subclinical abnormalities in glucose metabolism that are not detectable early in pregnancy. These factors may impair cardiac development even before diabetes is recognized in mothers who later develop gestational diabetes.^18^, ^19^, ^20^, ^21^, ^22^, ^23^, ^24^

The prevalence of pregestational diabetes in our study is lower than that reported in the general nongravid female population,^4^ but is similar to what is reported in pregnancy.^5^ This discrepancy may be attributed to the impact of diabetes on the endocrine reproductive axis^25^ and reduced fertility,^26^ potentially leading to an underestimation of the actual prevalence of diabetes in the general population. Additionally, there was a notable upward trend in the prevalence of pregestational diabetes and the incidence of gestational diabetes. This trend aligns with studies that have documented significant increases in both pregestational and gestational diabetes over the past decade.^27^ This increase is likely multifactorial, influenced by the obesity epidemic,^28^ poor nutrition,^29^ and physical inactivity,^30^ all of which are associated with an increased risk of these.^31^^,^^32^ Moreover, the increase in maternal age over the past decade^33^ may also contribute to the rising prevalence and incidence of pregestational^4^ and gestational diabetes.^34^

The reported incidence of CCHD varies significantly across studies, ranging from 0.05% to 0.13%,^1^^,^^35^, ^36^, ^37^ with our study reporting an incidence of 0.06%. This variation is likely due to multiple factors, including the population studied, study type, sample size, and methodology. Higher incidences of CCHD have been reported in Asia and Europe compared to North America.^37^^,^^38^ Smaller studies with robust diagnostic methods, such as echocardiography, for all study participants will detect milder defects and report higher incidences. However, the rarity of CCHD leads to statistical uncertainties with wide CIs, and generalizability may be limited.^1^ Conversely, larger studies provide more statistical power and are more generalizable but may fail to detect all CCHD.^1^ Our study sample represents the entire U.S. population with a high number of identified CCHD, increasing the accuracy and precision of our results. However, the data set utilized for this study records CCHDs diagnosed within the first 24 hours of life and may omit milder defects diagnosed later, which may underestimate the overall incidence.

Fetal echocardiography is recommended in cases of pregestational diabetes because of the significantly increased risk of CHD, as endorsed by both the American Heart Association and the Society for Maternal-Fetal Medicine. The risk of CHD in this population is estimated to be 3 to 5%, consistent with the increased OR of 4.33 observed in our study.^39^^,^^40^ In contrast, gestational diabetes has only been associated with a modest increase in CCHD risk, and the current evidence does not support routine fetal echocardiography in this group. Although subtle structural differences, such as increased interventricular septal thickness and larger left atrial area, have been reported, functional parameters are generally comparable between the gestational and nongestational diabetes groups.^41^ Our findings suggest that screening may be best individualized for higher-risk gestational diabetes cases. Furthermore, our study supports prior evidence that infants exposed to early onset preeclampsia have a higher prevalence of CHD than unexposed infants, potentially due to shared angiogenic pathways between placental development and fetal cardiac morphogenesis.^42^

In multivariable analysis, advanced maternal age was an independent risk factor for CCHD, and aging is known to increase the risk of developing diabetes,^34^ suggesting a possible cumulative effect of aging on CCHD. Furthermore, maternal sociodemographic factors—such as lower household income, limited educational attainment, and higher levels of material deprivation—have been independently associated with an increased risk of CHD in infants, even after adjusting for clinical and behavioral risk factors.^43^^,^^44^

However, race appeared to be a significant factor. Specifically, our findings indicate that the White race is associated with a higher risk of CCHD than the Black race. This aligns with previous research, such as the study by Ebeh et al, which similarly reported that both Asian and White races were linked to an increased risk of.^45^ The association between smoking and CCHD remains unclear. While some studies indicate an increased risk,^46^^,^^47^ others show no significant association.^48^, ^49^, ^50^ The American Heart Association reported that data were insufficient to determine the risk associated with smoking in CHD patients.^51^ In this study, maternal smoking correlated with a higher risk of CCHD, which contributes to narrowing this gap in knowledge. However, more prospective studies are required to further investigate this relationship.

### Strengths and limitations

The strengths of this study include the large sample size, low levels of missing data, and the inclusion of a broad array of variables, which allows for the analysis of factors associated with rare diseases, such as CCHD. These strengths, particularly the use of a comprehensive and large-scale national data set that covers the population nationwide, enhance the reliability and generalizability of the findings. Furthermore, this study sheds light on many factors associated with CCHD, potentially offering avenues for further research and intervention.

This study has several limitations that affect the interpretation of its results. Some forms of diabetes may have been misclassified. As half of U.S. pregnancies are unplanned, women without pregnancy intention may not undergo regular diabetes screenings, increasing the likelihood that cases labeled as gestational diabetes were actually undiagnosed preexisting diabetes due to late presentation. Additionally, reliance on database records limits access to glycemic control data, HbA1c levels, and treatment details, making it difficult to assess the severity of diabetes associated with CCHD. It is possible that mild diabetes has no association with CCHD, although this cannot be determined from the available data. Moreover, CCHD was documented using data recorded only within the first 24 hours of life, and this will miss milder cases of CCHD that may be diagnosed later and underestimate incidence rates. Terminations and stillbirths were excluded, and with advances in fetal cardiac diagnosis during the study period, some severe cases may have been detected prenatally and electively terminated. The likelihood of such detection, and thus the probability of being captured in our data set, may vary by timing of presentation, socioeconomic status, education level, and glycemic control, potentially influencing the observed association between diabetes and CCHD. Other important risk factors for CHD, such as family history of CHD and underlying genetic conditions, were not available in the database and, therefore, could not be included in our analysis. The absence of these variables is an inherent limitation of this data set. Despite controlling for numerous variables, the possibility of residual confounding exists, as factors such as detailed dietary habits, environmental exposures, and medications may not be fully accounted for.

## Conclusions

Our analysis revealed a strong association between maternal diabetes and the incidence of CCHD. Pregestational diabetes markedly increases the risk of CCHD, underscoring the critical need for stringent glycemic control and preconception counseling for women with diabetes. Gestational diabetes also poses a significant risk, but to a lesser extent, suggesting that glucose management throughout pregnancy is vital. Our findings advocate enhanced prenatal care strategies and targeted public health interventions to reduce the burden of CCHD, particularly in diabetic populations.

Perspectives **COMPETENCY IN MEDICAL KNOWLEDGE:** Pregestational diabetes confers over a fourfold higher risk of cyanotic CHD, underscoring the need for preconception metabolic control.

**TRANSLATIONAL OUTLOOK:** Targeted preconception counseling and intensive glycemic control for women with preexisting diabetes, combined with early metabolic screening and fetal echocardiography, may help reduce the incidence of cyanotic CHD.

## Funding support and author disclosures

Dr Gulati is supported by contracts from the National Heart, Lung, and Blood Institutes nos. N01-HV-068161, N01-HV-068162, N01-HV-068163, N01-HV-068164, grants U01 HL064829, U01 HL649141, U01 HL649241, K23 HL105787, K23 HL125941, K23 HL127262, K23HL151867, T32 HL069751, R01 HL090957, R03 AG032631, R01 HL146158, R01 HL146158-04S1, R01 HL124649, R01 HL153500, U54 AG065141, General Clinical Research Center grant MO1-RR00425 from the 10.13039/100000097National Center for Research Resources, the 10.13039/100006108National Center for Advancing Translational Sciences Grant UL1TR000124, 10.13039/100000005Department of Defense grant PR161603 (CDMRP-DoD), and grants from the Gustavus and Louis Pfeiffer Research Foundation, Danville, NJ, The Women’s Guild of Cedars-Sinai Medical Center, Los Angeles, CA, The Ladies Hospital Aid Society of Western Pennsylvania, Pittsburgh, PA, and QMED, Inc, Laurence Harbor, NJ, the Edythe L. Broad and the Constance Austin Women’s Heart Research Fellowships, Cedars-Sinai Medical Center, Los Angeles, CA, the Barbra Streisand Women’s Cardiovascular Research and Education Program, Cedars-Sinai Medical Center, Los Angeles, CA, The Society for Women’s Health Research, Washington, DC, the Linda Joy Pollin Women’s Heart Health Program, the Erika Glazer Women’s Heart Health Project, the Adelson Family Foundation, Cedars-Sinai Medical Center, Los Angeles, CA, Robert NA. Winn Diversity in Clinical Trials Career Development Award (Winn CDA), and the Anita Dann Friedman Endowment in Women’s Cardiovascular Medicine & Research; and has received consultant fees/honoraria from Esperion and Medtronic Inc, unrelated to this work. This work is solely the responsibility of the authors and does not necessarily represent the official views of the National Heart, Lung, and Blood Institute, the National Institutes of Health, or the U.S. Department of Health and Human Services. All other authors have reported that they have no relationships relevant to the contents of this paper to disclose.
